# Dysregulation of immune response in PCOS organ system

**DOI:** 10.3389/fimmu.2023.1169232

**Published:** 2023-05-05

**Authors:** Jingxuan Wang, Tailang Yin, Su Liu

**Affiliations:** ^1^ Reproductive Medicine Center, Renmin Hospital of Wuhan University, Wuhan, China; ^2^ Shenzhen Key Laboratory of Reproductive Immunology for Peri-implantation, Shenzhen Zhongshan Institute for Reproduction and Genetics, Shenzhen Zhongshan Urology Hospital, Shenzhen, China

**Keywords:** polycystic ovary syndrome, immune cells, cytokines, ovary, endometrium, vaginal microorganisms

## Abstract

Polycystic ovary syndrome (PCOS) is the most common reproductive endocrine disorder affecting women, which can lead to infertility. Infertility, obesity, hirsutism, acne, and irregular menstruation are just a few of the issues that PCOS can be linked to. PCOS has a complicated pathophysiology and a range of clinical symptoms. Chronic low-grade inflammation is one of the features of PCOS. The inflammatory environment involves immune and metabolic disturbances. Numerous organ systems across the body, in addition to the female reproductive system, have been affected by the pathogenic role of immunological dysregulation in PCOS in recent years. Insulin resistance and hyperandrogenism are associated with immune cell dysfunction and cytokine imbalance. More importantly, obesity is also involved in immune dysfunction in PCOS, leading to an inflammatory environment in women with PCOS. Hormone, obesity, and metabolic interactions contribute to the pathogenesis of PCOS. Hormone imbalance may also contribute to the development of autoimmune diseases. The aim of this review is to summarize the pathophysiological role of immune dysregulation in various organ systems of PCOS patients and provide new ideas for systemic treatment of PCOS in the future.

## 1 Introduction

Polycystic ovary syndrome (PCOS) is a common endocrine and metabolic disorder in women of childbearing age, which is closely related to female infertility, with an incidence of 5% -15% (1). The diagnosis of PCOS is a diagnosis of exclusion and should present with at least two of the three main symptoms: 1) clinical and/or biochemical hyperandrogenism (HA); 2) ovulatory dysfunction (OD); and 3) polycystic ovarian morphology (PCOM) (2). Clinical manifestations like insulin resistance (IR), obesity, hirsutism, and acne may also be present (3). Women with PCOS are more likely to develop type II diabetes, endometrial cancer, underlying cardiovascular disease, mood disorders, and depression. Additionally, multiple pregnancies, abortions, preeclampsia and pregnancy-induced hypertension, gestational diabetes, and other problems are more likely to occur in PCOS women (4). Currently, the etiology of PCOS remains unknown, as its symptoms are complex and diverse and cannot be completely cured clinically (5).

Primordial follicles consist of an oocyte resting at diplotene stage in meiosis I diplotene and a single layer of flattened anterior granulosa cells surrounding it. The majority of primordial follicles are more likely to stay dormant until death than to be activated (6). Activated primordial follicles form primary follicles, at which point pregranulosa cells will transform into a single layer of cuboidal granulosa cells (GC). The transformation of main follicles into secondary (preantral) follicles occurs with GC proliferation, differentiation, and oocyte growth. At the same time, theca cells begin to form around the outer granulosa cells and can produce androgens and serve as raw materials for estrogen production by GC (7). Accumulation of follicular fluid between GCs increases in response to estrogen and follicle-stimulating hormone, and follicular enlargement is called antral follicle (7). Atresia develops in the majority of antral follicles, and only antral follicles that react to follicle-stimulating hormone (FSH) and luteinizing hormone (LH) are likely to be selected for ovulation (7, 8). Due to the high expression of LH receptors in a subset of antral follicles that line the follicular wall, preovulatory LH spikes activate these follicles, causing them to ultimately ovulate the dominant follicle (9). PCOS is an important syndrome causing anovulation. Women with PCOS have larger-than-normal ovaries and more than 12 follicles that range in size from 2 to 9 millimeters (10). PCOS is characterized by an elevated density of small preantral follicles compared to normal ovaries (11), arrested follicular maturation with accumulation of follicular fluid and dilatation of antrum. Follicles gradually expand, apoptosis occurs in the GC layer and finally atresia occurs, resulting in the disappearance of GC in the follicular wall and the appearance of thin-walled cysts. The causes of ovarian folliculocytosis in PCOS patients are very complex and have been shown to be related to factors such as abnormal anti-mullerian hormone (AMH) secretion by GC, excessive androgen production by membranous cells, and insufficient FSH secretion leading to follicular maturation failure (12).

Regarding the pathogenesis of PCOS, recent studies have suggested that it is associated with genetic and environmental factors, intrauterine environment, endocrine, immune and metabolic dysfunction (13). Hyperandrogenism, obesity and IR interact in PCOS and are involved in immune disorders and systemic inflammation in PCOS (14). Obesity, as a metabolic disease, occurs in nearly 50% of women with PCOS and is one of the causes contributing to chronic low-grade systemic inflammation (15). BMI has already been shown to correlate with endometrial proliferation in women with PCOS. In PCOS patients, BMI is positively correlated with expression of endometrial marker of proliferation Ki-67 (MKI67) (16). Numerous studies have recently concentrated on the impact of chronic inflammation and low-grade inflammation on immunity, with hyperandrogenism playing a significant role in the emergence of immunological problems in PCOS. The prevalence of PCOS is strongly correlated with a number of inflammatory factors, including IL-6, TNF-α, IL-1, IL-18, IL-17, and inflammasomes (17). Additionally, PCOS was associated with a considerably higher proportion of various immune cell subsets (16). A low grade chronic inflammatory state in PCOS patients is caused by the accumulation of numerous inflammatory cells and multiple inflammatory cytokines (18). This article reviews the literature on the relationship between PCOS and immune cells and cytokines and their metabolic effects in various organ systems of the female body, and explores the immune response mechanism of PCOS.

## 2 Method

In this review, a literature search was performed in PubMed, Elsevier, and Wiley Online Library, including literatures published in English and available up to March 2023. The following key word were used for the search alone or in combination: polycystic ovarian syndrome (PCOS), obesity, insulin resistance, hyperandrogenism, inflammation, immune regulation, androgens, estrogens, cytokines, macrophages, monocytes, dendritic cells, natural killer cells, vaginal microorganisms, intestinal microorganisms, nonalcoholic fatty liver disease, autoimmune thyroid disease, subclinical hypothyroidism, adrenal androgens, dehydroepiandrosterone, dehydroepiandrosterone sulfate, COVID-19, obstructive sleep apnea. Literatures were selected for review based on their titles and abstracts which were relevant to the topic. The references of the articles correlating to this review were further searched and selected.

## 3 The impact of sex hormones on immune responses in women

By boosting the amount of immune cells that are in circulation and regulating the generation of cytokines in the body, sex hormones have an impact on the immune system (19). Typically, androgens have anti-inflammatory effects and can suppress immune cell activity (20). For example, androgen ablation increases the number of mature dendritic cells (mDCs) as well as expression of dendritic cell (DC) costimulatory markers in lymph nodes (21). Androgen causes a significant decrease in cell-surface toll-like receptor 4 (TLR4) expression in macrophage-like cell lines (22). In addition, androgens can also regulate adaptive immunity in humans by inhibiting Th1, Th2 and Th17 activity, but inducing Treg activity (23). However, the anti-inflammatory effects of androgens are not absolute. Hyperandrogenism in PCOS may change inflammation by influencing macrophage numbers and phenotypes. Higher M1 (inflammatory) and M2 (anti-inflammatory) macrophage ratios could be observed in 5α-dihydrotestosterone (DHT) -treated rat ovaries and PCOS female ovaries (24). Androgen receptor (AR) is expressed by normal skin, fibroblasts, epithelial cells, and macrophages in acute trauma (25). It is reported that castrated rats treated with androgens before urethroplasty have a prolonged inflammatory phase during healing, with upregulation of macrophages and TNF-α levels in urethral wounds (26). Estrogen has a more complex regulatory effect on the immune system (27, 28). Because estrogen levels fluctuate throughout the menstrual cycle, estrogen can suppress pro-inflammatory pathways at high levels during periovulation and indicate pro-inflammatory pathways when levels fall to the early follicular phase (29). Through the estrogen receptor α (ER α), estrogen can take role in the activation of the macrophage immunophenotype. Estrogen influences macrophage metabolic remodeling, enabling macrophages to cooperate with various activation pathways in different microenvironments (28). When estrogen levels are physiological, the immune system responses more strongly to bacterial endotoxins. Contrarily when levels are supraphysiological, the capacity of macrophages to bind lipopolysaccharide (LPS) is enhanced. It potentially leads to a more severe inflammatory response after bacterial infection (28). This reflects the dual effects of estrogen on the immune system in different microenvironments. Specifically, in autoimmune diseases like systemic lupus erythematosus (SLE), estrogen stimulates the expression of IL-6, which drives naive CD4 cells to differentiate into Th17 cells and inhibits TGF β of IL-6, which drives naive (30). In SLE, activation of IFN (α or γ) signaling upregulates estrogen receptor α (ERα) expression and stimulates target gene expression. Elevated levels of estrogen and IFN-α engage positive feedback loops that further intensify the inflammatory response in SLE (30, 31).

## 4 The impact of obesity on immune system

Obesity is a metabolic systemic disorder that affects metabolic homeostasis and causes low-grade inflammatory responses (32). It has been demonstrated that immune system deficiencies are related to obesity. A large number of neutrophils, M1 macrophages, and T cells could be observed in adipose tissues (33). Adipose tissue macrophages (ATM) play a dominant role in participating in systemic inflammatory responses. Increased secretion of monocyte chemoattractant protein-1 (MCP-1/CCL2) and leukotriene B4 (LTB4) by adipocytes promotes migration and infiltration of macrophages. Macrophages and adipocytes that accumulate abundantly in adipose tissue secrete adipose inflammatory cytokines TNF-α, IL-6, and IL-1β, activating the nuclear factor kappa-B (NF-κB) pathway to produce large amounts of inflammatory factors. Leptin secreted by adipose tissue is also one of the causes involved in immune disorders in obese individuals (34). In addition, adipose tissue was enriched for large numbers of CD4^+^ T cells as well as IFN-γ secreted by them. Macrophages and adipocytes overexpressing class II major histocompatibility complex (MHC II) and costimulatory molecules (e.g., CD80 and CD86) in adipose tissue act as antigen-presenting cells (APCs) to promote CD4^+^ T cell proliferation and Th1 differentiation and produce excessive IFN-γ in adipose tissue (35). Furthermore, leptin secreted by adipocytes can stimulate Th1 cells to secrete IFN-γ in excess and induce MHC II overexpression in adipocytes, further aggravating the inflammatory response (36).

## 5 PCOS-related immune dysregulation in the female reproductive system

The immune system orchestrates the HPO axis to participate in normal female physiological processes, such as ovulation, fertilization, pregnancy, and embryo implantation. Immune cells like neutrophils, T cells, and macrophages are recruited to the ovaries during fertilization and migration (37). It has been proved that luteal production and regression are influenced by macrophage, T cell, and granulocyte infiltration (38). Endometrial immune environment is important to the maintenance of normal pregnancy. On the one hand, Endometrial immune cells protect against pathogen infiltration, on the other hand, the immune system exerts immune tolerance functions and contributes to the implantation of embryos and normal pregnancy (39). The composition and function of the microorganisms in the genital tract are also influenced by mucosal local immunity. Immune regulation issues in PCOS-affected women further promote the development of chronic inflammation (40). The mechanisms involved in immune as well as metabolic disorders in PCOS in the reproductive system will be investigated below.

### 5.1 PCOS-related immune dysregulation in the ovary

Ovarian tissue is mainly composed of ovarian parenchyma and ovarian stroma. The parenchyma is composed of ovarian follicles, whereas the stroma is composed of immune cells, blood vessels, nerves, lymphatic vessels, and ovarian-specific components (41). Macrophages, dendritic cells, neutrophils, eosinophils, mast cells, B cells, T cells, and natural killer (NK) cells are among the immune cells found in the ovary. Ovarian immune cells have multiple functions, including phagocytosis and antigen presentation, remodeling of tissues by proteolytic enzymes, and secretion of soluble signals, including cytokines, chemokines, and growth factors (41, 42).

PCOS patients are in a state of chronic low-grade inflammation, which may trigger a cascade of events that further promote the content of ovarian androgens and affect ovulation. PCOS patients present with an abnormal androgen response to gonadotropin-releasing hormone (GnRH) stimulation leading to ovarian androgen overproduction (43). Immune cells and cytokines interact with androgens resulting in disruption of ovarian immune balance in PCOS. For example, González’s results showed that mononuclear cells (MNCs) entering the ovary may cause a local inflammatory response that stimulates ovarian androgen production in women with PCOS (44). Li et al. showed that the IFN-γ levels were decreased in PCOS rats induced by dehydroepiandrosterone (DHEA). It is possible that DHEA inhibited proliferation and promoted apoptosis of ovarian granulosa cells and down-regulated IFN-γ expression (43).

Follicles represent the basic functional units of the ovary (7). Follicular fluid (FF) which is composed of follicular cell secretion and theca vascular exudate, which contains gonadotropins secreted by the pituitary gland and steroid hormones secreted by the ovary, changes with follicular development (45). In recent years, there have been plenty of studies demonstrating that abnormal inflammation can alter normal ovarian follicular dynamics, leading to impaired oocyte quality, anovulation, and associated infertility (46). DCs are specialized innate immune cells that sense danger signals, absorb and process antigens, and transmit them to T lymphocytes (47). Prior to impending ovulation, DC are important components of bone marrow-derived leukocytes in the microenvironment of mature oocytes and their abundance and maturity may be related to ovarian function in women with PCOS (48). Evidence has shown that the mean fluorescence intensity (MFI) of human leukocyte antigen DR (HLA-DR) expression reflects a positive correlation between DC maturity and ovarian response as measured by serum E2 levels on the day of human chorionic gonadotropin (hCG) administration. E2 production measured 48 h prior to oocyte retrieval was associated with the presence of more mature DCs, while this association was strengthened when analyzing patients undergoing *in vitro* fertilization (IVF) due to male factor infertility (i.e., normal ovarian function). This suggests that maturity of DC in FF is positively correlated with gonadotropin response and may favor an aseptic inflammatory process leading to ovulation in follicles (48). In addition, the percentage of CD11c^+^ HLADR^+^ DCs was significantly lower in FF of PCOS patients than in normal controls. It is also possible that reduced DCs may influence the activation of Th17/Th1 cells, leading to failure of dominant follicle selection and developmental processes (49).

Th cells play a role in adaptive immunity by producing cytokines.Th1 mainly secrete IL-2 and IFN-γ to promote cellular immunity, and Th2 mainly secrete IL-4 to regulate humoral immunity (50). Local coordination of T lymphocytes impacts survival of granulosa cells and embryo quality in female ovaries. Changes in T cell distribution can promote follicular survival either by providing trophic growth factors or inhibiting adverse immune activity, or conversely by transmitting cytotoxic signals to induce oocyte or granulosa cell death and promote follicular regression (51). Early studies have found that memory T lymphocytes in the theca layer of PCOS ovaries are reduced compared to non-PCOS ovaries (52). Qin, et al. showed that Th1 cytokines (IFN-γ, IL-2) production in FF lymphocytes was significantly higher in PCOS patients than in controls, and Th1 cytokines predominate in FF of PCOS patients as analyzed by flow cytometry. On the contrary, the production of Th2 cytokines (IL-4, IL-10) was not statistically significant between the two groups, suggesting that the imbalance of Th1/Th2 cell ratio may affect egg quality and ovulation (53). Li et al. showed that the percentage of total CD4^+^ T cells and CD8^+^ T cells was significantly decreased while the expression of PD-1 was increased in FF of the infertile PCOS patients. The failure of dominant follicle selection and development was caused by higher PD-1 levels, which further supported the pathogenic function of local T cell imbalance in PCOS (54).

Granulocyte colony-stimulating factor (G-CSF) is a cytokine that stimulates neutrophil proliferation and differentiation, which is mainly secreted by granulosa cells before ovulation. G-CSF produced by granulosa cells may recruit leukocytes to the thecal layer during ovulation to accelerate ovulation (55). G-CSF concentrations in follicular fluid and serum were also significantly higher in PCOS patients than in controls. The neutrophil count and neutrophil/leukocyte ratio of PCOS patients were significantly higher than those of controls, further supporting the theory of chronic inflammation in PCOS (55, 56). Other studies have shown that IL-18 levels in FF of PCOS patients are higher than those in controls, especially the level of IL-18 in FF of overweight PCOS patients is significantly higher than that in normal weight PCOS patients (57). More studies showed increased levels of IL-1β, IL-6, and TNF in FF of PCOS patients (17, 58, 59). The reason for this is that inflammatory cytokines in follicular fluid alternately alter the follicular microenvironment, activating the NF-κB inflammatory pathway. The inflammatory cascade may affect granulosa cell proliferation, inhibit oocyte maturation, and aggravate ovulatory dysfunction more severely (58) (**Table 1**).

**Table 1:** The immune cells and cytokines in follicular fluid of PCOS.

Immune cells	Related immune cells or cytokines	Function	References
CD11c^+^HLA-DR^+^DCs	Th17, Th1	Failure of dominant follicle selection and developmental processes	(49)
Th1	IFN-γ, IL-2	Affection on egg quality and ovulation caused by Th1/Th2 cell ratio imbalance	(53)
Th2	IL-4, IL-10		
CD4^+^ T cells, CD8^+^ T cells	PD-1	Failure of dominant follicle selection and development	(54)
Immune factor			
G-CSF	Neutrophil	Chronic inflammation in PCOS	(55, 56)
IL-18, IL-1β, IL-6, and TNF-α		Activation of NF-κB inflammatory pathway	(17, 57–59)

### 5.2 PCOS-related immune dysregulation in the endometrium

The human endometrium is a steroid-dependent tissue, and hormonal changes during the ovulatory cycle can affect the growth and remodeling of endometrial cell components and tissues (60). In addition to guarding against infections, female reproductive system immune cells also enable embryo implantation and establish immunological tolerance to sperm and embryo/fetus (61). As steroid hormones (progestins, androgens, and estrogens) change with the menstrual cycle, it has been demonstrated in an increasing number of studies that immune cells and inflammatory factors have an impact on the reproductive system’s ability to function. Steroid hormones either directly or indirectly affect the expression of chemokines IL- 8 and MCP-1) as well as the survival and apoptosis of resident endometrial cells (stromal cells, epithelial cells, and endothelial cells) and immune cells (39, 62). CD56^+^ uterine natural killer cells (uNK), CD68^+^ macrophages and CD8^+^ cytotoxic T lymphocytes are all common endometrial/decidual immune cells (63), and They regulate endometrial function by releasing cytokines, such as IL-15, IL-10 and IFN-γ (64).

The endometrium of women with PCOS has continuous estrogen exposure during both the proliferative and secretory phases, while diminished progesterone action during the secretory phase is likely to impair endometrial receptivity and lead to long-term endometrial hyperplasia, bleeding, and cancer (65). In addition to sex hormones, metabolic disorders and chronic inflammatory conditions caused by obesity and hyperinsulinemia promote oxidative stress imbalance in PCOS endometrium and affect progesterone receptor activity in PCOS endometrium. Women with PCOS had a poorer reaction to progesterone than did women without the condition, and they had thicker surface epithelium and more stromal cells than women without PCOS, but considerably fewer blood vessels overall (66). uNK cells are one of the most important immune cells of human uterine leukocytes. The main endometrial NK cells are CD16^−^ NK cells, accounting for 70-80% of secretory endometrial lymphocytes (67). The percentage change of uNK with hormones during the menstrual cycle may play a key role in implantation and maintenance of pregnancy, especially the number of decidualized endometrium is further increased in the first trimester (67, 68). Female sex hormones appear to regulate uNK recruitment indirectly by modulating chemokine and interleukin expression. It has been shown that the percentage of CD56^+^/CD16^−^ NK cells and CD56^bright^/CD16^−^ NK cells decreased in the late secretory endometrium of PCOS women, while the proportion of CD3^+^ lymphocytes significantly increased. Meanwhile, CD56^+^ and CD56^bright^ NK cells were reduced, the expression of IL-15, IL-18, and CXCL10 was also significantly lower in PCOS than that of the control group, which may be related to chronic oligo-ovulation or hyperandrogenism in PCOS patients (68). CD68^+^ macrophages are seen in the endometrium throughout the menstrual cycle, particularly in the late luteal phase (69). Endometrial macrophages may be involved in the onset of menses, repair and remodeling of the functional layer of the endometrium, and play an important role in the preparation of a receptive endometrium during the “window of implantation” and endometrial decidualization (61). Macrophages within the endometrium have been identified as an important source of proinflammatory and chemotactic factors that specifically express role-specific markers at different stages of the menstrual cycle (70). It has been shown that endometrial CD68^+^ macrophages and CD163^+^ M2 macrophages are significantly increased in PCOS patients, which may be related to insulin resistance and the release of inflammatory factors in PCOS (71). One of the main endocrine features of PCOS is hyperandrogenism, while androgens can induce TNF-α production by macrophages. Recent studies have observed that the proliferative endometrial TNF-α level is significantly increased in PCOS patients (72, 73). Therefore, the increased number of macrophages in the endometrium of PCOS patients may be responsible for the increased TNF-α (72). DCs are mainly located in the functional and basal layers of the endometrium and are broadly classified according to their developmental pathways: plasmacytoid DCs (pDCs) and Myeloid DCs. Myeloid DCs have a high correlation with the endometrium and can be divided into immature DCs (iDCs) and mDCs according to maturation status (74). In response to foreign antigens or inflammatory signals, mDCs present antigens together with MHC molecules to T cells, effectively initiating adaptive immunity (71, 75). Studies have shown that the increased percentage of endometrial CD1a^+^ iDCs, CD83^+^ mDCs in normal weight PCOS patients, and confirmed that the dysfunction of DCs may be related to the pathogenesis of PCOS. Granulocyte-macrophage colony-stimulating factor (GM-CSF) can promote DC and endometrial macrophage maturation (71). Studies have found that GM-CSF down-regulation in endometrial stromal fibroblasts (eSF) of women with PCOS, which may be associated with poor endometrial receptivity and DC cell migration (76). Endometrial T cells include CD4 Th1, Th2 CD8, Treg, and Th17 cells, mainly located in the decidual stroma and glandular epithelium (77). Decidual tissues had the highest concentration of CD8^+^ T lymphocytes. Endometrial CD8^+^ T cells are elevated in PCOS patients, indicating that the immune environment of the endometrium is altered and T cells may be involved in endometrial immunoregulatory mechanisms in PCOS (71). Previous studies have also shown that high levels of MCP-1 increased the terminal differentiation of CD4^+^ T cells into Th2 cells, while the basal level of MCP-1 was also increased in PCOS patients. It indicates that T cells may play a role in the pathogenesis of the condition (76).

**Figure 1:**
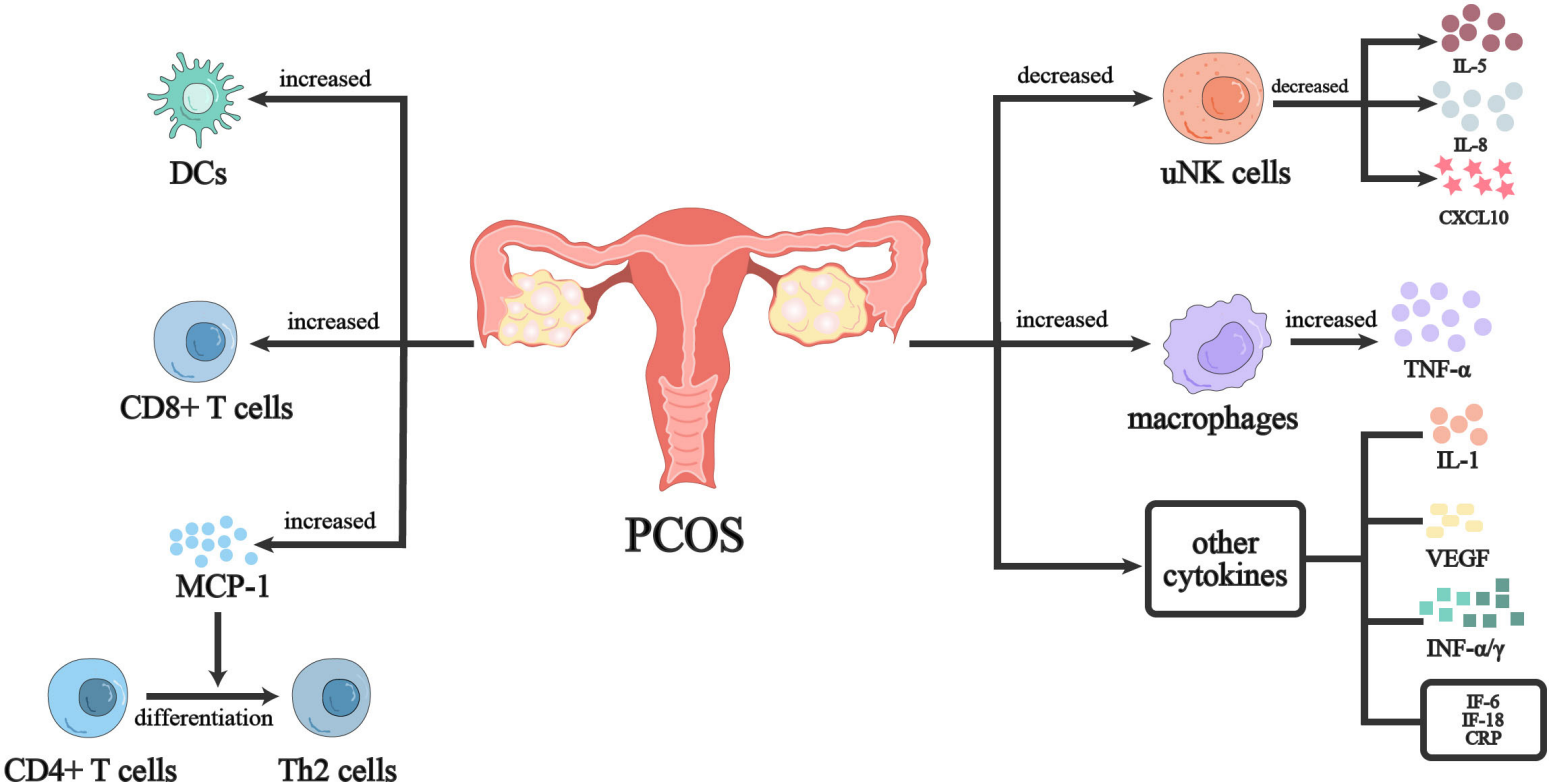
Immunoregulation of endometrium in PCOS women. PCOS women have a imbalanced immune environment in the endometrium. Both the proliferation and differentiation of T cells in PCOS women’s endometrium as well as the proliferation of innate immune cells such uNK cells and dendritic cells are influenced. At the same time, cytokines secreted by immune cells are dysregulated. Further evidence of the inflammatory milieu in PCOS women’s endometrium came from the upregulation of inflammatory molecules like TNF, CRP, and IL-6.

Cytokines and chemokines in the endometrium also affect the endometrial immune microenvironment in PCOS patients. At present, IL-1 and vascular endothelial growth factor (VEGF) are closely related to endometrial receptivity. The expression levels of IL-1 and VEGF in endometrium of PCOS rats were significantly lower than those of the control group, suggesting that the endometrial receptivity of PCOS rats was significantly lower than that of the normal control group (78). It has been found that the key elements of TLR -mediated NF-κB signaling pathway were dysregulated in endometrial tissue of PCOS women, and the expression of TLR4 protein was increased in the endometrium. IRF-7 and NF-κB signaling may be activated and TRL4 positively regulated by hyperandrogenism, which may also boost the expression of cytokines like IFN-α and TNF-α in the endometrium (79). In addition, the inflammatory environment in the endometrium of women with PCOS is also thought to be associated with the overexpression of other cytokines, such as IL-6, IL-8, IL-18, and CRP (76, 80, 81) (**Figure 1**).

### 5.3 PCOS-related immune dysregulation in the vaginal mucosa

Healthy female vagina is colonized by multiple normal microbial and fungal groups, which are divided into beneficial microorganisms and opportunistic pathogens inhabiting the vaginal environment, with lactobacilli as the dominant genus (82, 83). The effects of estrogen and progesterone on vaginal epithelial cells, PH, sexual activity, menstruation, and antibiotic usage are the key factors affecting the vaginal microbiome (83). The body benefits from the homeostasis of vaginal bacteria since they are a crucial part of the microenvironment of the reproductive tract (40). Increasing evidence suggests that the composition of a woman‘s vaginal microbiota can significantly impact her sexual and reproductive health, including her risk of adverse delivery outcomes, including miscarriage and premature delivery, as well as infection with HIV and other sexually transmitted pathogens (84–86). The stratified squamous epithelial cells that cover the mucus layer are part of the vaginal ecosystem, along with vaginal bacteria, neutrophils, macrophages, classical dendritic cells, Langerhans cells, NK cells, T and B lymphocytes, and other innate and adaptive immune cells (86). The vagina contains many immune-related cells and receptors that detect pathogenic organisms primarily through microbial motif pattern recognition of pattern recognition receptors (PRRs), such as TLRs or dectin-1 receptors (40). Additionally, vaginal defense is aided by mannose-binding lectin (MBL), vaginal antimicrobial peptide (AMP), immunoglobulin A, and immunoglobulin G (IgA, IgG) (40).

The impact of PCOS on women’s vaginal health is mainly reflected in the disruption of homeostasis of the vaginal microenvironment. Hong’s study showed that the vaginal microbiome is associated with clinical manifestations of PCOS, such as acanthosis nigricans, intermenstrual bleeding, etc. When compared to healthy women, PCOS patients with high testosterone levels had a higher relative abundance of *L. crispatus* and a lower relative abundance of *L. iners*. On the other hand, their relative abundance of *Mycoplasma* and *Prevotella* was significantly higher than that of controls (87). Another study also demonstrated that *L. crispatus* and *L. iners* populations were more sensitive to testosterone levels in women with PCOS (88). Tu showed that there was a significant decrease in lactobacilli in lower genital tract (LGT) organisms in PCOS patients, while *Gardnerella vaginalis* was significantly enriched in both the vagina and cervix of PCOS patients, in addition to several potential pathogens including *Gardnerella*, *Prevotella*, *Veillonellaceae*, *Streptococcus*, and *Dialister* species (89). *Gardnerella*, *Prevotella*, and other species produce sialidase, IgA protease, and short-chain fatty acids, which lead to local IgA inactivation and, respectively, improve their adherence to epithelial cells, evade antibody-mediated inhibition, and modulate the immune environment (90, 91). Prevotella also contributes to activation of Th17 immune responses via APCs, promotes increases in cytokines such as IL-23A, IL-6, IL-1A, and IL-1B that promote Th17 immune responses, and recruits and activates Th cells in inflamed vaginal mucosa (92). Bacterial products of certain anaerobes have been shown to induce the production of short-chain fatty acids from pro-inflammatory cytokines by TLR stimulation, dendritic cell activation and maturation, and by producing specifically short immune cell migration, apoptosis, and phagocytosis (93). This suggests that disturbances in vaginal microbial homeostasis in PCOS may be associated with impaired mucosal immunity.

## 6 PCOS-related immune dysregulation in the cardiovascular system

Increasing evidence suggests that women with PCOS are at increased risk for coronary artery disease (CAD) and cardiovascular disease (CVD) (15, 94, 95). Insulin resistance is one of the most important pathogenesis of PCOS and an important cause affecting cardiometabolism in women with PCOS (96). IR increases a woman’s risk of CVD by being linked to a number of cardiometabolic disorders, including dyslipidemia, hypertension, diabetes mellitus, and metabolic syndrome (97). Oxidative stress and chronic inflammation have been implicated in the pathogenesis of IR in PCOS, including increased reactive oxygen species (ROS) production by peripheral blood leukocytes, activation of leukocyte-endothelial interactions, and increased levels of the pro-inflammatory transcription NF-κβ, as well as pro-inflammatory cytokines and C-reactive protein (98). The low-grade chronic inflammatory state of PCOS is likely to provide a pathophysiological basis for the development of CVD, particularly the development of atherosclerosis. Microparticles (MPs) are subcellular vesicles that can be released practically by any cell and range in size from 100 to 1000 nm. They are a major indicator for identifying cardiometabolic risk in PCOS (99). MPs derived from leukocytes (LMPs) may originate from neutrophils, monocytes/macrophages, and lymphocytes, alter endothelial function, participate in coagulation and platelet activation, and promote the recruitment of inflammatory cells into the vessel wall, contributing to atherosclerotic lesion progression (100). A study showed higher levels of LMPs in PCOS patients, suggesting that MP may be closely associated with the development of atherosclerosis in PCOS patients (100). CRP plays a role in and triggers atherothrombotic processes as one of the recognized markers that can forecast cardiovascular events. In CAD patients with low-grade or persistent inflammation, CRP can be used in combination with the biomarkers MCP-1 and galectin 3 to predict recurrent events (101). MCP-1 can recruit monocytes to the vessel wall via its C-C chemokine receptor type 2 (CCR-2) on monocytes as a chemokine (102). Hu et al. showed that serum concentrations of CRP and MCP-1 were significantly higher in PCOS patients compared with controls. The possible mechanism is that elevated CRP levels promote monocyte accumulation in the atherogenic arterial wall by increasing monocyte chemotactic activity in response to MCP-1 (103). A meta-analysis showed that women with PCOS had significantly higher levels of CRP, Hcy, PAI-1 antigen, PAI-1 activity, VEGF, ADMA, AGEs, and Lp (a). Although it is unclear how IL-6 and TNF-α are related to CVD events in PCOS, these inflammatory factors are probably significant indicators for predicting CVD in PCOS (104, 81).

## 7 PCOS-related immune dysregulation in the digestive system

### 7.1 Intestine

The gut microbiota (GM) is a complex community with physiological roles such as constituting the gut barrier, stimulating the immune system, and anabolism (105). The impact of PCOS on the gastrointestinal tract is mainly reflected in the disruption of gut microbial diversity and homeostasis, while the gut microbiota affects the development of the immune system and regulates immune mediators, which in turn affect the intestinal barrier (106).

According to growing evidence showing the GM in PCOS patients differs from those of healthy women, suggesting that microbial imbalance or “dysbiosis” in the gut may contribute to the pathology of PCOS (107–109). Qi et al. showed that bile acids are involved in regulating IL-22 production to affect ovarian function in PCOS. IL-22 mRNA, tauroursodeoxycholic acid (TUDCA) levels, and GATA3 levels were significantly decreased in mice transplanted with stool from individuals with PCOS. In addition, serum IL-22 levels in PCOS-like mouse models also decreased. Similar to the mice research, PCOS patients had significantly lower serum and follicular fluid levels of IL-22 than in controls. Because mice preferentially conjugate bile acids with taurine, humans predominantly use glycine. Intestinal and serum IL-22 levels and intestinal GATA 3 mRNA levels increased in PCOS-like mouse models after glycodeoxycholic acid (GDCA) administration. Secretion of IL-22 protein and Il22 mRNA levels were significantly increased in group 3 innate lymphoid cells (ILC3s) cultured *in vitro* in the presence of TUDCA or GDCA. The reason for this is that bile acids induce IL-22 secretion by intestinal ILC3s via the GATA 3 signaling pathway, which in turn improves the PCOS phenotype (110). Lindheim et al. showed a significantly lower abundance of *Tenericutes* in the gut of PCOS patients compared to healthy women and a negative correlation with total blood lymphocyte counts (107). More studies have shown that LPS produced by intestinal flora has endotoxin effect, and LPS-binding protein can bind to TRL4 on the surface of innate immune cells and mediate PCOS-related inflammatory response, further aggravating IR symptoms in PCOS patients (111, 112).

### 7.2 Liver

Nonalcoholic fatty liver disease (NAFLD) encompasses a spectrum of diseases ranging from simple steatosis without inflammation or fibrosis to nonalcoholic steatohepatitis (NASH), to fibrosis, cirrhosis, and finally hepatocellular carcinoma (113, 114). The etiology of PCOS and NAFLD share the same features: they are all strongly associated with IR, hyperandrogenism, and obesity (113, 115, 116). In recent years, increasing evidence suggests an association between NAFLD and PCOS, but there are few studies on immunomodulation in NAFLD in PCOS women. Increased plasma levels of IL-6 and TNF-α have now been demonstrated in NAFLD and NASH patients, and increased production of TNF-α and IL-6 by peripheral blood mononuclear cells from NASH patients (117). Mohammadi et al. showed that the levels of IL-6 and CRP were significantly increased in PCOS rats, while the levels of IL-6 and CRP were significantly decreased in curcumin-treated PCOS rats (118). Increases in IL-6 and TNF-α are likely related to depletion of DCs located in the central and periportal veins aggravating aseptic inflammation in the liver and enhancing TLR4 and TLR9 activity and expression in innate effector cells (119, 120). Chen et al. showed that nine differentially expressed genes (DEGs), TREM1, S100A9, FPR1, NCF2, FCER1G, CCR1, S100A12, MMP9, and IL1RN, were significantly upregulated in PCOS and NAFLD, whereas these DEGs have been demonstrated to be associated with immune and inflammatory responses (121).

## 8 PCOS-related immune dysregulation in the endocrine system

PCOS is one of the common reproductive endocrine system diseases in women. In this section, we only discuss dysregulated immune response in other endocrine system rather than reproductive system.

### 8.1 Thyroid

Recent research has demonstrated that autoimmunity, particularly autoimmune thyroid disease (AITD) and subclinical hypothyroidism (SCH), may be strongly linked to PCOS etiology. AITD, the most widespread autoimmune antibody disease, is more prevalent in women with PCOS than in non-PCOS women and is the most frequent cause of hypothyroidism or subclinical hypothyroidism in the adult population (122–125). AITDs include Hashimoto‘s thyroiditis (HT) and Grave’ s disease (GD). The pathogenesis of GD and HT may be related to triggering of T cell- and B cell-mediated immune responses, which may eventually develop generalized hypothyroidism (125, 126). More women may have only higher antibody levels without significant thyroid dysfunction, leading to SCH (126). Several studies have demonstrated that autoimmune antibodies such as anti-TPO, anti-TG, anti-TSH are significantly elevated in women with PCOS (127, 128). The cause of AITD in women with PCOS is likely to be associated with hyperandrogenism. Androgen levels rise *in vivo* as a result of increased GnRH and LH pulse frequency in women with PCOS (126). Excessive androgens can enhance T suppressor cell activity or promote Th1 responses, and Th1-mediated autoimmunity leads to thyroid cytolysis and hypothyroidism, leading to HT (125). Aromatase converts androgens to estrogens, which causes compensatory increases in estrogen levels. Additionally, binding to estrogen receptors appears to have proliferative effects on B lymphocytes, T lymphocytes, and macrophages (126). Women with PCOS tend to have inadequate progesterone secretion, estrogen increases IL-6 expression in T cells, and the absence of progesterone suppression may lead to overstimulation of the immune system, making these patients more susceptible to autoimmune diseases (128).

### 8.2 Adrenal gland

The ovary is the main source of androgens in women with PCOS. Indeed, it has long been shown that adrenal androgen secretion is also increased in PCOS (129). DHEA and dehydroepiandrosterone sulfate (DHEA-S) are the two primary adrenal androgens in PCOS women. Acne vulgaris, as one of the common dermatologic manifestations of PCOS, has also been shown to be associated with higher concentrations of dehydroepiandrosterone sulfate (130). Peripheral conversion to testosterone nevertheless contributes to hyperandrogenism despite minimal adrenal androgen activity (131). It is reported that adrenal androgen (AA) has been reported in 20% to 30% of PCOS patients (132). A meta-analysis showed that DHEA levels were significantly higher in women with PCOS compared to healthy controls (133). Corticosteroid-steroidogenesis may therefore be an independent factor for hyperandrogenism in some women with PCOS and may be a genetic, stable trait (134). DHEA as well as DHEA-S have been demonstrated to have immunomodulatory functions in human cytological experiments, mainly affecting immune cell numbers by modulating cytokine levels (135). DHEA is involved in ovarian immune regulation and affects the balance of Th1 and Th2 immune responses in the ovary. It enhances Th1 responses while weakening Th2 responses by reducing the release of IL-2 and IL-10 (i.e., Th2-related cytokines) and the expression of the activation marker CD69 on CD4^+^ T cells, resulting in a new balance of Th1/Th2 immune responses (136). This shows that adrenal androgens may be associated with the immunological response in PCOS, but further research is needed to determine the precise mechanism.

In addition, according to a study, there is a unique clinical phenotype of PCOS. This phenotype is characterized by age-specific hyperandrogenism, but the patient‘s hyperandrogenism initially decreases to the normal range by approximately 35 years of age. The hyper-/hypoandrogenic PCOS phenotype (HH-PCOS) is known for having comparatively low androgen levels compared to the traditional PCOS phenotype (137). Gleicher et al. found that women with the HH-PCOS phenotype showed an activated immune system, particularly a strong association with anti-thyroid autoimmunity in the form of anti-TPO antibodies (138). Whereas adrenal autoimmunity is highly associated with other autoimmune abnormalities, antiadrenal and antithyroid autoimmunity is frequently observed in the same patient (123). Insufficient cortisol (C) production in the zona fasciculata can be detected in HH-PCOS. It is tempting to speculate that the putative autoimmune attack on the adrenal gland is not limited to decreased androgen production in the zona reticularis, but also affects the adjacent zona fasciculata (139). This suggests that HH-PCOS is likely an immune/inflammatory disease and is associated with autoimmunity.

## 9 PCOS-related immune dysregulation in the other system

On account of the global outbreak of novel coronavirus pneumonia, COVID-19 has been increasingly investigated in PCOS. PCOS patients may have a higher susceptibility to COVID-19, which is also increased by the presence of comorbidities such as NAFLD, obesity, alterations in the gut microbiome (140, 141). Hyperandrogenism in PCOS may be one of the main causes of high susceptibility to COVID-19. Androgens modulate immune responses, decrease NK cell activity, reduce TRL4 expression on macrophage surfaces, and also suppress pro-inflammatory responses by reducing extracellular signal-regulated kinase and leukotriene formation in neutrophils (142). In PCOS mice, elevated androgens upregulated SARS-CoV-2 receptor angiotensin converting enzyme 2 (ACE2), which acts synergistically with host transmembrane protease serine 2 (TMPRSS2) to increase SARS-CoV-2 viral entry into tissues (141, 143). Vitamin D can regulate the immune function of the body and play an important role in inducing macrophage differentiation, inhibiting the maturation of dendritic cells and blocking the adaptive response to antigen presentation, and enhancing the development of Treg cells (144). Furthermore, vitamin D can also down-regulate the synthesis of pro-inflammatory factors (IL-1, IL-6, IL-12, TNF-α and IL-17) and increase the expression of anti-inflammatory factors (IL-1). Macrophage activation correlates well with the severity of COVID-19 (145). Vitamin D has been linked to COVID-19 in a growing number of studies (146–148), although further research is needed to determine its significance for PCOS patients. One study showed that vitamin D was significantly lower in women with PCOS and was negatively correlated with BMI. Women with PCOS had higher levels of the pro-inflammatory macrophage-derived biomarkers CXCL5, CD163, and matrix metalloproteinase 9 (MMP9), but CD200 expression was lower. Pro-inflammatory expression of these macrophage-derived proteins was linked to obesity. CD80 was identified as one of the specific markers of activated Treg in circulation (149), whereas IL-12 induced Th1 cell differentiation and stimulated IFN-γ synthesis (150). Vitamin D deficiency has been associated with decreased CD80, IFN-γ, and IL-12 in PCOS in women with PCOS (151). These findings imply that one of the potential high-risk variables contributing to PCOS patients’ susceptibility to COVID-19 infection may be vitamin D deficiency.

Obstructive sleep apnea (OSA) is also an obesity-related disorder and is generally more prevalent in men than in women (152). OSA is characterized by repeated partial or complete airway collapse that may lead to intermittent hypoxia. Intermittent hypoxia further contributes to oxidative imbalance, producing reactive oxygen species, numerous cytokines such as IL-2, IL-4, IL-6, lipid peroxidation, and free DNA (153). There has long been much evidence that women with PCOS have a higher prevalence of OSA than the normal population (154–156). Although androgen excess may influence the prevalence and severity of OSA in both men and women, it does not necessarily cause OSA in women with PCOS because androgen levels in this population are still lower than those in men (157, 158). Nevertheless, hormones may play a protective role in the development of OSA. For instance, IL-6 secretion is elevated in sleep apnea, yet estrogen can inhibit IL-6 secretion (157). The true predisposing factors for OSA in women with PCOS may be IR and obesity (152). Obesity/insulin-resistance may be the main cause of sleep apnea, which in turn may accelerate these metabolic abnormalities because of the gradual rise of cytokines, such as IL-6 and TNF-α (159).

## 10 Discussion

PCOS has a range of effect on organ system in addition to the female reproductive system. Immune system function is impacted by hormonal disorders, and PCOS patients suffer low-grade chronic inflammation due to abnormal cytokine secretion, immune cell dysfunction, and hormonal disorders. At the same time, the etiology of chronic inflammation in PCOS is also influenced by obesity and metabolic disorders (particularly insulin resistance). In a vicious circle involving hormones, obesity, and IR, inflammatory cells and inflammatory markers accumulate up in PCOS women and disrupt the immune microenvironment of PCOS. In addition to having an impact on the reproductive system of PCOS patients, where it affects ovulation, endometrial receptivity, and folliculogenesis, abnormal immune function also contributes to the dysfunction of other systems in PCOS women. Patients with PCOS exhibit microbial dysbiosis, CVD, NAFLD, and OSA, all of which are closely associated with immunological regulation. Compared to healthy women, women with PCOS are even more susceptible to inflammatory illnesses and COVID-19.

One of the PCOS diagnosis criteria and one of its most prevalent symptoms is hyperandrogenism. Sexually dimorphic immunoreactivity typically uses androgens as anti-inflammatory hormones and estrogens as pro-inflammatory hormones. Furthermore, considering women experience menstruation cycles, changes in sex hormones have an impact on the growth of female follicles and ovulation. However, androgens are not just straightforward anti-inflammatory hormone in PCOS. In PCOS patients with hyperandrogenism, DEGs are highly enriched in immune and inflammatory responses (160). Nevertheless, there is controversy over how androgens affect fat tissue in PCOS. There is a stronger correlation between visceral and abdominal obesity in women with PCOS, as well as a significantly higher incidence of NAFLD. Obesity is a chronic inflammatory disorder in which necrotic adipocytes attract inflammatory cells and release inflammatory cytokines like TNF-α; PCOS is also associated with higher levels of M1 macrophages and inflammatory cytokines like TNF-α and IL-6. More significantly, obesity by itself is not a PCOS diagnostic indicator. Moreover, there is evidence that an excess of androgen is not responsible for chronic inflammation in PCOS but instead has anti-inflammatory benefits when obesity is present (161).

From the viewpoint of organs and tissues from the systemic system, we outline the pathogenic function of immune imbalance in PCOS women in this review. It is more convincing to demonstrate that PCOS is a systemic metabolic syndrome as well as an illness of the reproductive system. The review does, however, have some constraints. The manuscript is solely based on the author’s collection of literature, and the opinion that was eliminated has some subjectivity in the author’s opinion. The significance of immune cell and immune factor imbalance in PCOS has been summarized in earlier research. This manuscript begins with immune cells and immune factors as well. However, it concentrates more on the relationship with systemic organs and examines how obesity, hormones, and metabolic disorders interact with immune cells and immune factors in PCOS inflammation.

**Figure 2:**
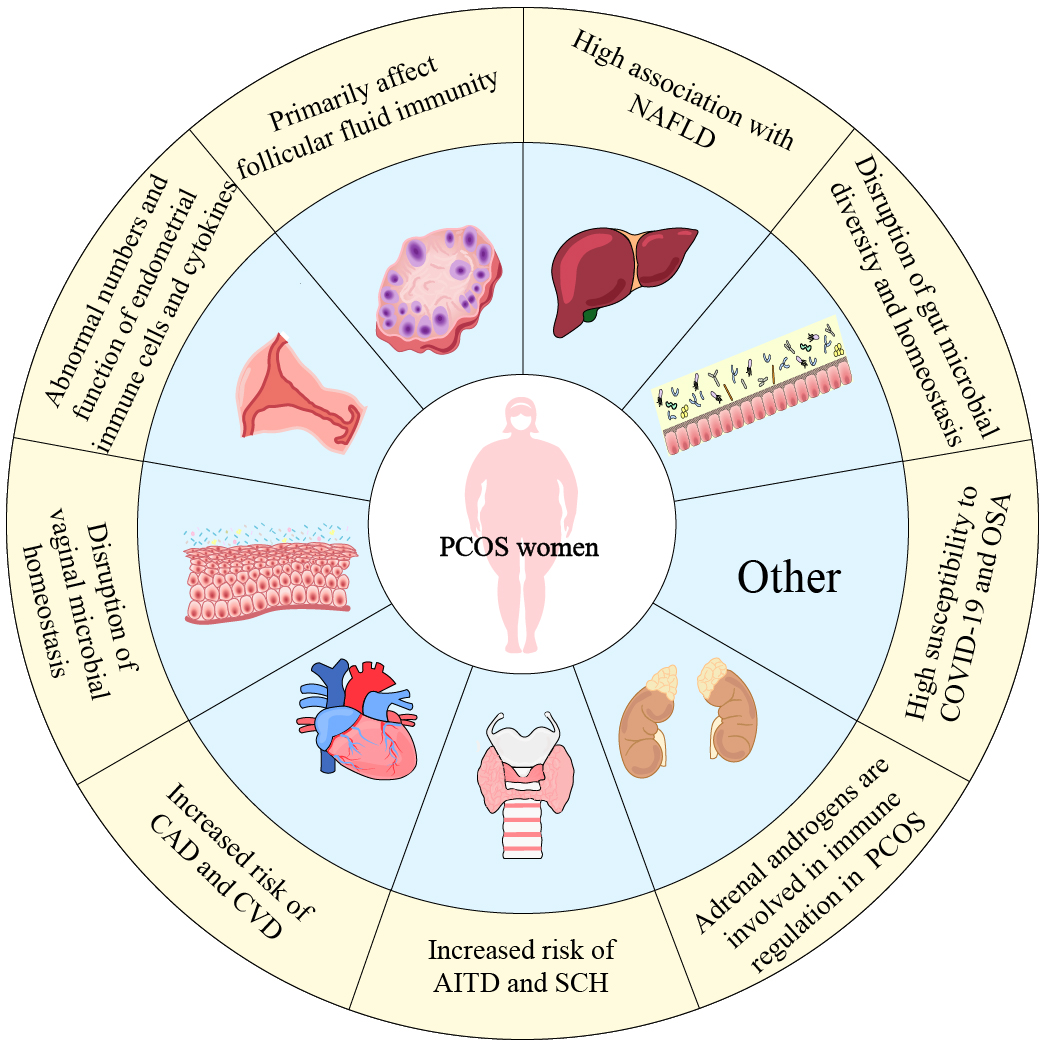
The dysfunction of organ systems related to immune dysregulation in PCOS women. This figure summarizes immune dysfunction in PCOS women in various systems. Not only the female reproductive system, the impact of PCOS is even reflected in the cardiovascular, intestinal, thyroid and other organs.

In addition to helping to evaluate chronic low-grade inflammation, understanding the immune cell phenotype and cytokine expression in PCOS patients can help predict the development of other diseases. Consequently, figuring out the pathogenic function of immune regulation in PCOS is crucial for both treatment and preventing further complications in the future (**Figure 2**).

## Author contributions

JW and TY conceived the original idea of the manuscript. JW drafted the manuscript and develop the figures. JW, TY and SL revised the first version of the manuscript. SL provided critical feedback and helped revise the manuscript. All authors contributed to the article and approved the submitted version.
